# Analysis on ecological characteristics of Mississippian coral reefs in Langping, Guangxi

**DOI:** 10.1038/s41598-022-22081-8

**Published:** 2022-11-16

**Authors:** Dayong Yang, Honglun Chang, Xiao Liu, Peng Wan, Liming Shen

**Affiliations:** 1 School of Transportation and Geomatics Engineering, Shenyang Jianzhu University, Shenyang, 110168 China; 2 Experimental and Practical Teaching Center, Hebei GEO University, Shijiazhuang, 050031 China; 3 Shandong No. 3 Exploration Institute of Geology and Mineral Resources, Yantai, 264011 China; 4 Shandong No. 8 Exploration Institute of Geology and Mineral Resources, Rizhao, 276800 China

**Keywords:** Ecology, Ecology, Environmental sciences

## Abstract

Several Late Viséan-Serpukhovian coral reefs were identified in Langping, Tianlin. They provided an opportunity to investigate paleo-environments suitable for the development of reef-building communities and the construction of coral reefs in Langping. In this paper, part of the reef-building environmental and the ecological characteristics of coral reefs then were elaborated by analyzing the development settings, palaeogeography, sedimentation of reefs, the response to hydrodynamic conditions of reef-building corals, effects of disturbance and non-reef-building organism on reef communities, and the influence of coral morphology on reef development. It is considered that the sedimentary environment of Langping in Late Viséan-Serpukhovian is suitable for the development of benthic communities. The current appearance of reefs is determined by both coral populations ecological characteristics and reef-building environment.

## Introduction

**Figure 1:**
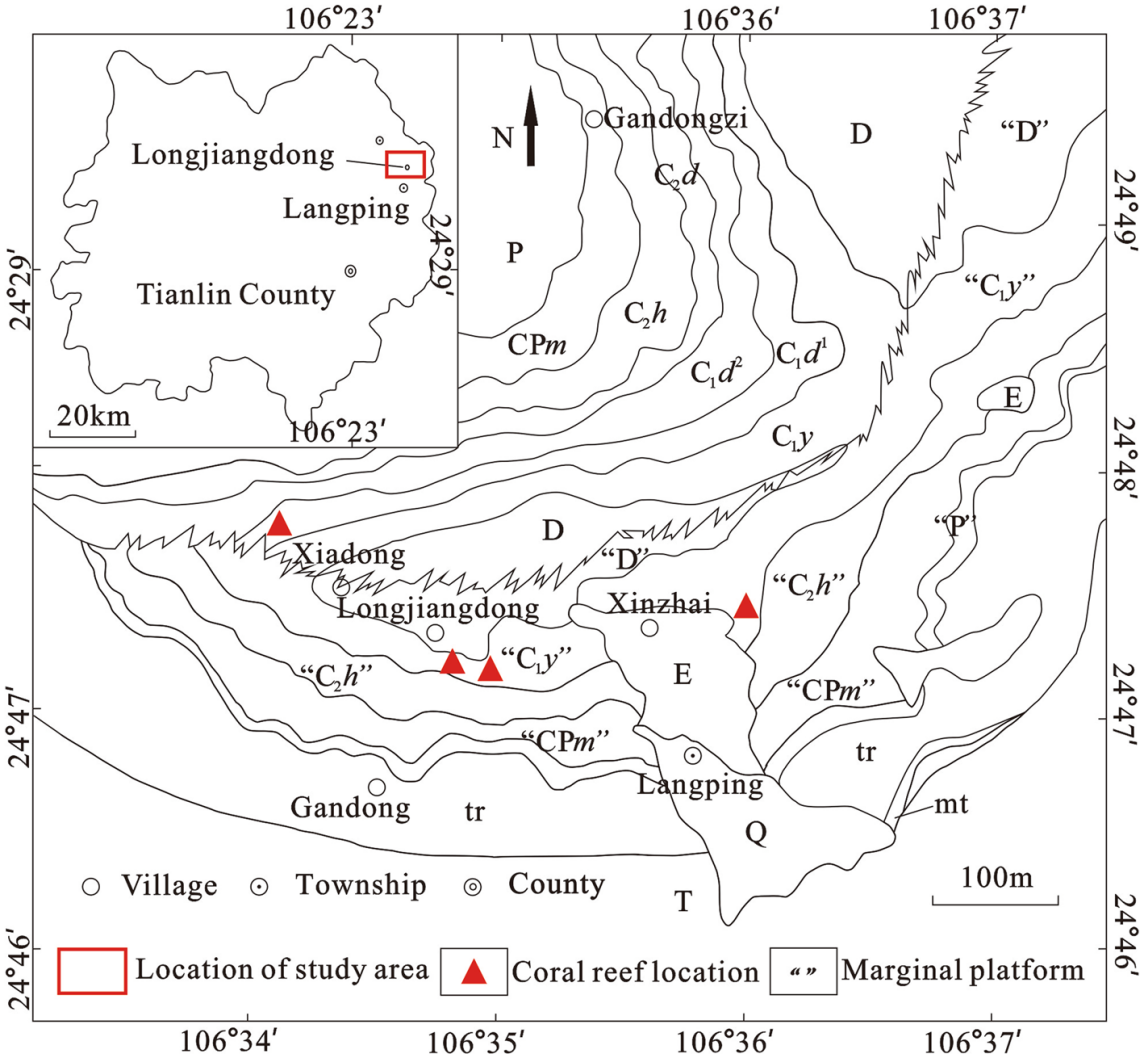
Geological map with coral reef outcrops in the study area. This figure was modified by the author from 1:5000 Regional Geological Survey Report by Geological Survey Institute of Guangxi Province and Administrative Map of Tianlin County. The software is CorelDRAW (vision 2022) and the URL link is https://www.coreldraw.com/cn/.

Early Carboniferous coral reefs in southern China were identified in the Yaoyunling formation and Du'an Formation in Xiadong village, Longjiangdong Village and Xinzhai Village, Langping Township, Tianlin County, Guangxi Province (Fig. 1). These reefs were of low degree maturity, with relatively few reef-building species, single reef-building way and short duration of construction. However, the benthic communities and ecology of the reefs differ from other contemporaneous reefs of Carboniferous^1,2^. The discovery of these coral reefs was significant for supplementing the evolution sequence of Carboniferous reefs and studying the development rules of the whole Carboniferous reef ecosystem^1,3^. The environmental setting of reef-building communities was one of a series of factors affecting the specific appearance and evolution of reefs, which was the foundation in controlling the overall characteristics of reefs, and played a leading role during the process of reef development and evolution^4–6^. Various factors affecting the specific appearance of the Mississippian shallow sea reefs were deduced by investigating reef ecological environments in the Langping and the ecological characteristics of the reef communities.

Although the ecological conditions for reef development are generally similar^6–8^, with regard to temperature, water quality, light, nutrition and oxygen, there are obvious differences in these environmental factors and ecological features among reefs with diverse appearances. These will result in significant diversity among reefs directly or indirectly^9–12^. Fang and Hou^13^ and Gong's research team^2,10,14–16^ have conducted significant research on many reefs in Langping. The palaeogeographic environment of Langping is favourable for reconstruction of the benthic communities, by combining lithofacies recognition, biological fossil identification, and sedimentary microfacies analysis^14,15^. Several of the reefs developed in the study area are rare late Viséan-Serpukhovian framework reefs with characteristics such as biodiversity, construction patterns, continuity, maturity and size that are different from synchronous reefs in other areas^2,15,16^. These characteristics reflect the unique environmental conditions and ecology of biological communities in the study area. Previous studies on the reef in Langping focused on paleontology, sediment and deposition. This paper takes a two-pronged approach, namely environmental factors and biotope characteristics, to explain the factors and influencing mechanisms that shape the specific appearance of these ancient reefs.

## Sampling and methods

Field observation, identification, description, measurement, and photographing were carried out at four reef outcrops in Langping. On the basis of clearly observing the boundary of each reef, the exposed extension size of the reefs was accurately measured. The biological species and content, sediments were recorded in detail and photos were taken. About 100 representative samples of sediments and corals were collected from the base, center and roof of each reef, and corresponding data were recorded. Subsequently, more than 130 thin sections, 30 polished slabs surfaces and 200 photos were taken in the lab in preparation for microfacies paleoecological analysis. These samples now are stored in Department of Geology, Northeastern University, Shenyang, China.

## Reef overview

The coral reef outcrop in Xiadong Village were exposed in EW direction and developed into large reefs without obvious discontinuity or evidence of multiple growth cycles. The reef was about 260 m in length and 50 m in height. Main builders consist of colonial corals *Diphyphyllum* and *Siphonodendron* with obvious vertical changes in the distribution of reef-building coral assemblages. Three successive reef-building coral population or coral assemblage, *Diphyphyllum*, *Siphonodendron–Lithostrotion,* and *Diphyphyllum–Syringopora*, can be clearly identified from bottom to top.

The multi-layer reef in Longjiangdong Village could be divided into three layers, 27 m × 3.8 m, 16 m × 2.5 m, and 40 m × 9 m in length and thickness respectively extending nearly EW. *Diphyphyllum* constructed the framework for reef layers. Coral clusters were presented on various scales with maximum of 2 m × 3 m in height and width, most being vertical clusters.

The patch reef in Longjiangdong Village was smaller, with the outcrop 11.2 m long and 6.2 m thick. The reef builders were colonial *Diphyphyllum*, solitary *Caninia,* and colonial *Lithostrotionella*. The monomeric *Tetracoralla* eventually formed an upper layer of the reef 3–5 m wide.

The coral layer reef preserved in the east of Xinzhai Village was about 15 m long and 5–6 m thick. Colonial *Lonsdaleia* formed a framework at the base of the reef, overlaid by unidentified dendrimers and colonial coral *Antheria*. Solitary corals eventually formed the reef’s upper layer, with the thickness of 3–5 m.

## Results and discussion

Notwithstanding constraints on the amount of hard data, according to our integrated analysis, the developmental environment and ecology of reef communities have an important impact on the appearance of reefs.

### Analysis of environmental conditions for reef development

#### Settings of reef development

The F/F extinction event in Late Devonian caused the complete recession of the reef-building communities based on stromatoporoid-coral assemblages^7,17^. The Carboniferous is generally considered to be a sub-optimal period for the development of framed reefs. After the biological mass extinction, microorganisms and algae rebuilt new reef-building ecosystems^18,19^. Some short-term biological frame reefs developed with low diversity, limited reef-building organisms, small sizes, and restricted distribution^20^. Harsh climate and marine conditions occurred in the Mississippian, including extensive marine hypoxia, repeated glacial and interglacial climate changes, and frequent changes of sea level and seawater surface temperature, potentially hindering the recovery of Early Carboniferous metazoan reefs^7,21^.

Metazoans gradually began to participate in reef building in Early Viséan. A large number of biogenic structures formed by corals and bryozoans began to appear, including a small number of sponge reefs/mounds in the middle and late stage of Viséan. The richness and biodiversity of the Mississippian post-zoobenthic reefs flourished in the late Viséan during which corals, bryozoans, sponges, calcareous microorganisms, and some calcareous algae became the main builders^3^ and large-scale reefs could also be seen in some areas although most of the Viséan metazoan reefs were tabular or laminar. Thus, the metazoan skeletal reefs in the middle to late Viséan were considered to have been resurrected due to relatively warm climatic conditions and higher sea levels after a period of complete disintegration at the end of the Devonian and recession at the beginning of the Carboniferous^7^.

Consequently, the coral reefs in the study area were the products of shallow benthic communities thriving in relatively favourable conditions of Late Viséan-Serpukhovian, which was common for reef development at that time^7^. Thus, it is expected that more synchronous reefs would to be identified in southern China, or even in the study area in the future.

#### Paleogeography of reefs

**Figure 2:**
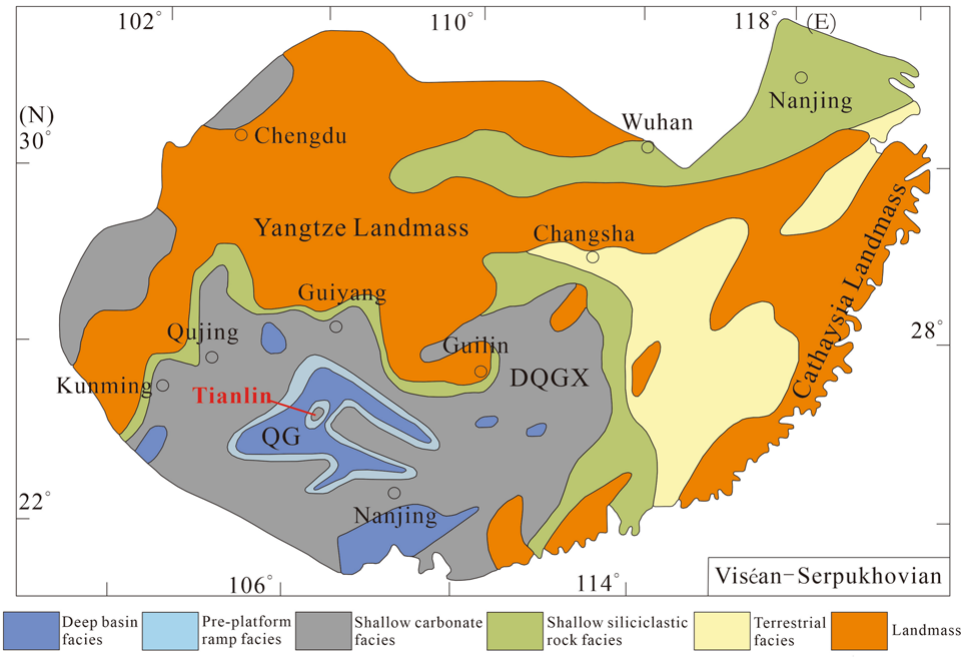
Paleogeographic map of southern China in Viséan-Serpukhovian (modified from Feng^23^, Yao^8^, and Maillet^24^). This figure was obtained from articles by Feng^23^, Yao^8^ and Maillet^24^ respectively. The author modified the picture with CorelDRAW (version 2022, and the URL link: https://www.coreldraw.com/cn/). *QG* Qian-Gui Basin, *DQGX* Dian-Qian-Gui-Xiang platform.

Langping is located in Dian-Qian-Gui Basin^22^ regionally (Fig. 2), in the eastern end of Tethys tectonic domain and at the interjunction of Tethys and Pacific structure globally. The Carboniferous Dian-Qian-Gui Basin is adjacent to the Tethys Basin. During the Early Carboniferous, the continent of Gondwana was close to the equator but was separated from the northern continent by the Tethys, where the tropical currents flowed freely from east to west. The benthic warm-water organisms were distributed widely with high abundance and diversity on both sides of the shallow shelf of Tethys.

Viséan-Serpukhovian ecosystems experienced dramatic climate changes and widespread glaciation^25^. However, the Viséan was also a key layer for a variety of biological structures, with abundant coral reefs and a high diversity of shallow benthic communities, peaking in the late Viséan. Newly discovered post-faunal reefs in Tianlin were mainly formed in the late Viséan-Serpukhovian period, which coincided with frequent sea level fluctuations and possible glacial changes. It seems counterintuitive that tropical coral communities developed during glacial period. However, recent studies suggest that the persistent warm ocean currents on the platform helped some coral species survive from Carboniferous glacial events^24^. While other areas of symbiotic reefs were poorly developed, Tianlin may provide an ecological sanctuary for corals associated with ocean currents^26^.

#### Sedimentation of reef development

According to the regional geological structure, the slope model for Langping paleocarbonate platform was obviously different from that of steep slope platform margin, which could be directly affected by waves. Langping palaeo-platform could be regarded as one of the small blocks (block fault barrier) separated from a large platform (continental margin sea basin)^13^. The relative positions of these blocks were crucial for the emergence and growth of reefs.

In situ development of mud-crystalline tuffs and muddy tuffs with weak hydrodynamic conditions is common in the Langping area, and evaporites are poorly developed. There were patch reefs and reef layers in different sizes in the wide intraclast beach, where obviously developed reef beach complexes were rare. The fragments of carbonate base broken by storm in the clastic beach haven’t been observed. The study area is considered to be gentle-slope open platform^27,28^ based on sedimentary characteristics. It suggests that the study area was far away from the margin of steep-slope platform that directly affected by waves, and more consistent with less energetic internal environments of gentle-slope platform.

On the vast platform of Langping gentle slop, deep water lead to low water energy. While in the coastal area, the water energy was relatively strong, thus coarse-grained bioclastic beach and a small amount of point reef could be developed. The beaches were irregular-shaped due to long term transportation and reformation effects of waves and water flow, showing low and gentle slope angles. Dispersed reef-beach complexes at the platform margin slightly impacted inner-platform seawater and the water flows smoothly^29^.

**Figure 3:**
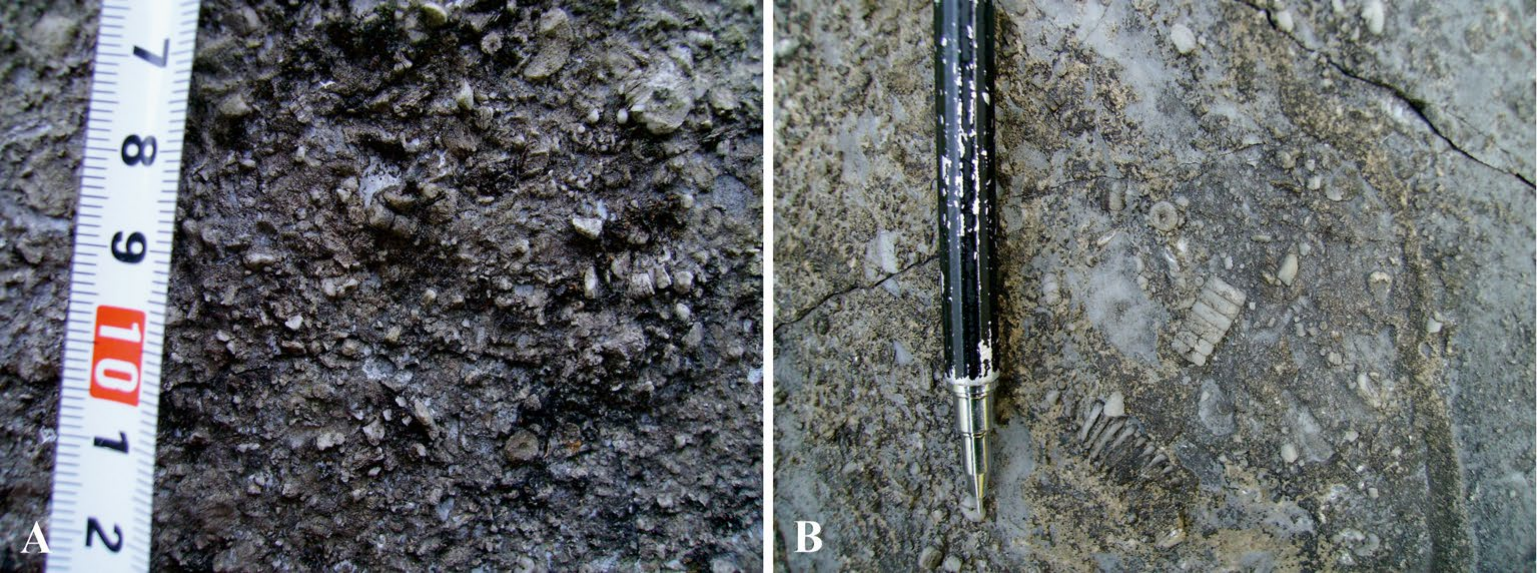
Clastic beaches in the Langping. Various clastic beaches developed in the study area. Diverse composition, fragmentation degree and sorting of the clast indicate different water conditions of formation. This figure is modified by the author from field photos with CorelDRAW (version 2022, and the URL link: https://www.coreldraw.com/cn/).

**Figure 4:**
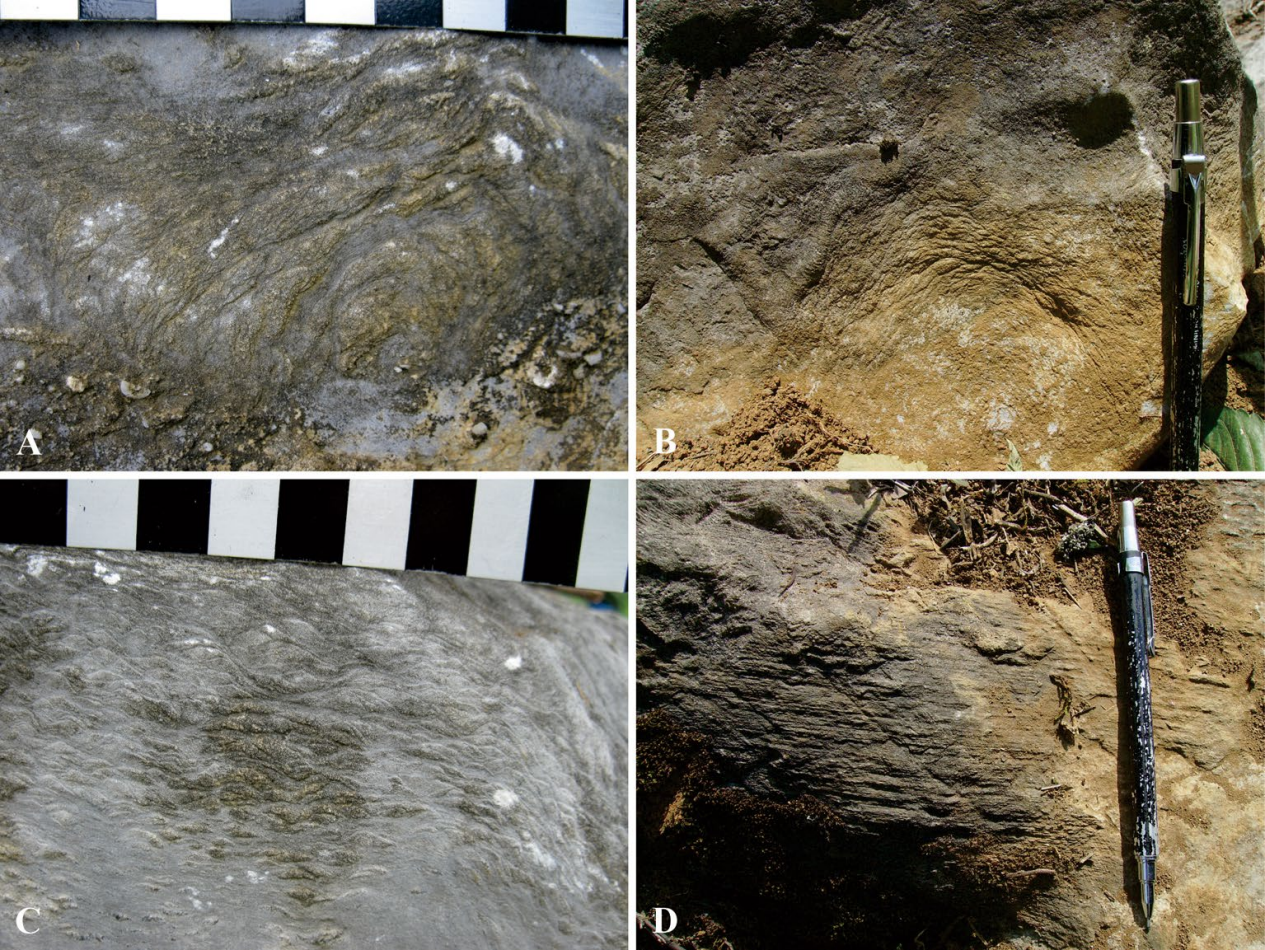
Algal bondstone in the Langping. Bondstone formed by various algaes living in still water. Morphology of bondstone correlates water environment and deposition of mud. Vast algal bondstones indicate deep water and high deposition rate. This figure is modified by the author from field photos with CorelDRAW (version 2022, and the URL link: https://www.coreldraw.com/cn/).

Therefore, it can be assumed that Langping reefs developed along the intertidal shallows of the terrace. The seawater around Langping carbonate platform in Late Viséan-Serpukhovian was relatively shallow while the water flow was strong. Remains of crinoids, brachiopods, a few foraminifera, and solitary corals were likely broken by strong currents, and deposited in situ with a small amount of gravels and lime-mud (Fig. 3). The clastic beach was unstable, suggesting large-scale wave-resistant structures could not be formed quickly^30^ due to insufficient cohesive and consolidating organisms. In addition, the circumferential impact of water in extensive terraces leads to mud-lime deposition, which is detrimental to most benthic organisms. However, bondstone was more likely to be formed by some binding algaes in the platform (Fig. 4). Therefore, neither the surrounding or the inner region of the platform could provide favourable living conditions for coral reefs to develop over for a long term. The gently sloping terrace environment of Lanping resulted in significant differences in growth size, wave resistance and reef-building capacity between corals in the study area and those on the edge of the steeply sloping terrace.

At the same time, the warm climate of the late Viséan-Serpukhovian, the good circulation of seawater around the Langping platform, and the abundant supply of oxygen and nutrients were a series of favourable conditions that facilitated the growth of reef-building corals, which led to uplifts being formed on clastic beach, including patch reefs and reef layers with certain sizes. These uplifts impeded waves and provided a protected nearshore environment, though they were much smaller than those developed at the steep-slope platform margin. The inhabitants on the beach could not resist strong waves. These rises were therefore known as reef-beach complexes and could only persist where waves and currents were mild^28^. They were essentially different from the framework coral reefs which developed on steep-slope platform margin that reflected changed hydrodynamic conditions, nutrient sources, reef sizes, and growth rates.

Another potentially favourable factor in the study area could be the deeper water area in the gentle-slope sedimentary environment, which could provide more stable conditions and reduce the damaging effects of global glacial events and large scale sea level fluctuations on reef-building communities^25^. The frequent fault activities in Dian-Qian-Gui Basin caused the rise and fall of equivalent sea level. More influence of sea-level fluctuations and hydrodynamic conditions would be exerted on Langping platform due to its small size. Furthermore, reef growth promoted by reef-building communities would be frequently disturbed. The sediments displaying evidence of multicycle sedimentation, different components, and diversely fragile clasts in the study area provided direct evidence of frequently changing environment.

Alternatively, the sedimentary environment of Langping platform provided conditions favorable for reef-building communities to develop and reefs to grow rapidly. These factors directly or indirectly determined the ecology of reef-building communities and the general appearance of reef development in the study area.

Overall, the environmental factor is the primary factor affecting the overall development trend of reefs.

### Inferred ecological characteristics of reef communities

#### Response of reef-building corals to hydrodynamic conditions

Hydrodynamic conditions are very important factors for reef development, which directly determine the abundance and distribution of each reef-building population and are key factors influencing sedimentation and reef growth, and was particularly evident at Langping. Evidence from the fossils suggested the reef-building corals were also changed in response (Table 1). The hydrodynamic condition changes during the development of reefs are inferred based on analysis of the vertical sediments and microfacies changes of coral reefs in the study area^31^. How these ancient reef-building corals adapted to hydrodynamic conditions was reconstructed combining the evolution of reef-building communities with the study.

**Table.1:** General situation of reef-building coral population in Langping.

Reef	Dimensions of reef-building corals	Population size
Xiadong coral reef	*Diphyphyllum* individuals are mainly 0.4–0.6 cm in diameter and 10–20 cm in length	*Diphyphyllum* clusters are large and some reaches about 2 m
	*Siphonodendron*'s cross-section shows round outline individuals about 0.5–1 cm in diameter, with generally small spacing	*Lithostrotion* and *Siphonodendron* clusters are generally 10 to dozen centimeters in diameter. The *Lithostrotion* clusters can reach nearly 2 m
	*Lithostrotion* individuals are 0.3–0.5 cm in diameter and closely arranged	*Syringopora* clusters are small and generally tens of centimeters
	*Syringopora* individuals are thin, and are connected by connective tubes, forming clusters	
Longjiangdong multi-layer reef	*Diphyphyllum* individuals are 0.4–1 cm in diameter and 10–20 cm in length	*Diphyphyllum* clusters are large, up to 2 m in height and 3 m in width
Longjiangdong patch reef	*Thysanophyllum* individual diameter is 0.5–0.7 cm	*Thysanophyllum* clusters showed a length and a height of tens of centimeters
	*Caninia* are generally 1–3 cm in diameter	*Caninia* scattered distribution
	*Lithostrotionella* individual ranges from 0.5 to 1 cm in diameters	*Lithostrotionella* clusters are generally tens of centimeters long
Xinzhai layer reef	*Lonsdaleia* individuals are about 1 cm in diameter and 5–20 cm in length	*Lonsdaleia* population generally formed in clusters with length and height of tens of centimeters
	The unidentified dendrimers are about 1 mm in diameter and several millimeters to several centimeters in length	Unidentified dendrimer population is several centimeters high and tens of centimeters long
	*Antheria* individuals are about 1 cm in diameter and grow closely	*Antheria* population is formed in clusters with tens of centimeters in length and height
	The solitary coral is 1–2 cm in diameter	Solitary corals grow in small-scale colonies, with clusters length and height of tens of centimeters

**Figure 5:**
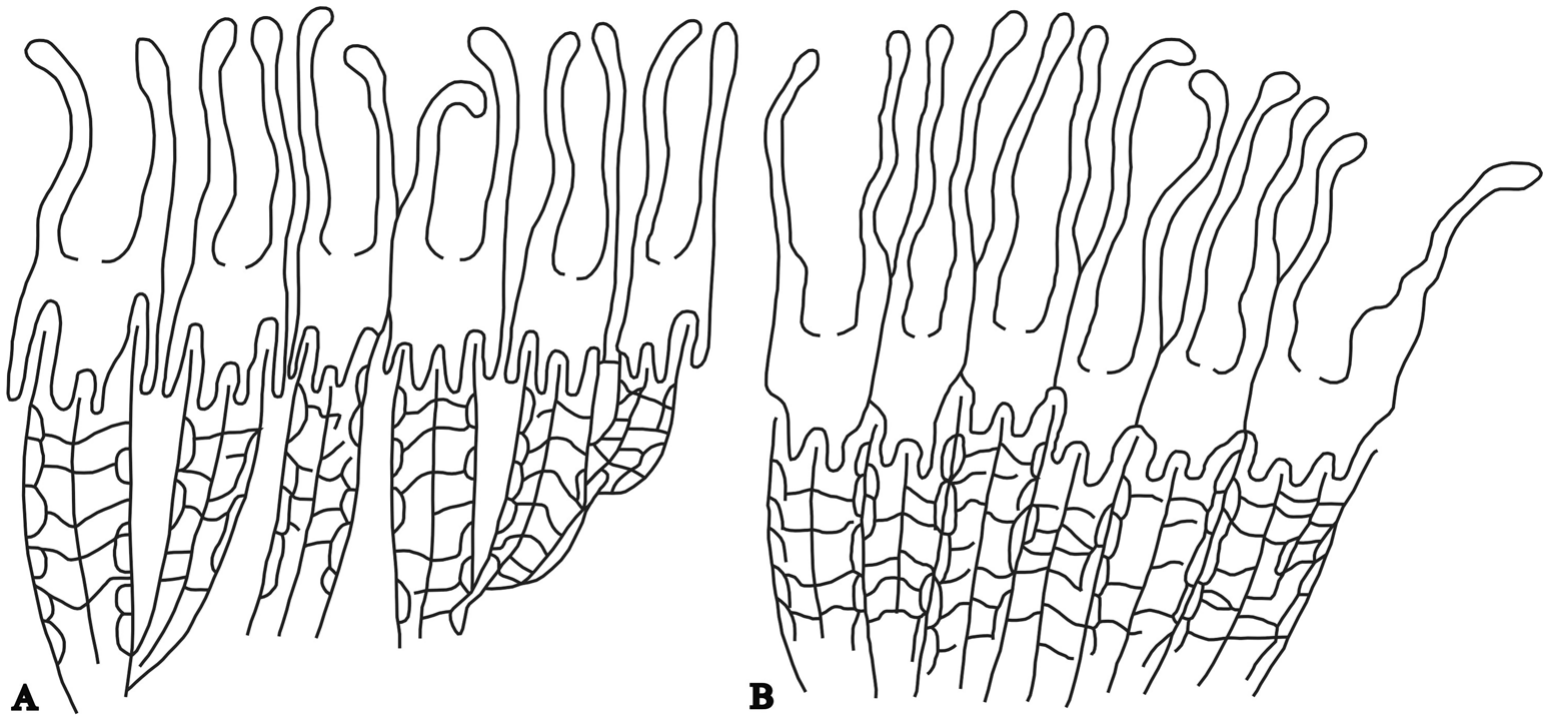
Sketch of coral cluster with upright growing morphology. Most reef-building corals in Langping grow vertically into cluster colonies. This type of morphology is very favourable for corals to get more living space and is important to reef-building. (**A**) Cluster coral individuals grow uprightly with certain distance between each other. (**B**) Polygonal columnar coral individuals grow closely to resist strong water flow. This figure is made by the author with CorelDRAW (version 2022, and the URL link: https://www.coreldraw.com/cn/).

The Xiadong coral reef started with colonization and expansion of *Diphyphyllum* on the bioclastic hard substrates^32,33^. They grew vertically into upright clusters (Fig. 5A) and were insensitive to more sediment in a relatively calm, turbid water environment^34^. The relatively dense clumped *Siphonodendron* and massive *Lithostrotion* (Fig. 5B) were better suited to the turbulent water environment, becoming dominant over time, with *Diphyphyllum* subordinate with the continuous increase of the water energy, as indicated by the characteristics of sediment particles from fine to coarse. After flourishing for a period, the *Siphonodendron*–*Lithostrotion* assemblage eventually waned, likely due to the failure to adapt to the increasing hydrodynamic conditions. *Diphyphyllum* had persisted combined with *Syringopora*, to maintain the growth of the reef. However, this assemblage subsequently declined as a result of strong hydrodynamic conditions and finally died out in response to continuous falling of sea level. Consequently, the reefs stopped developing.

**Figure 6:**
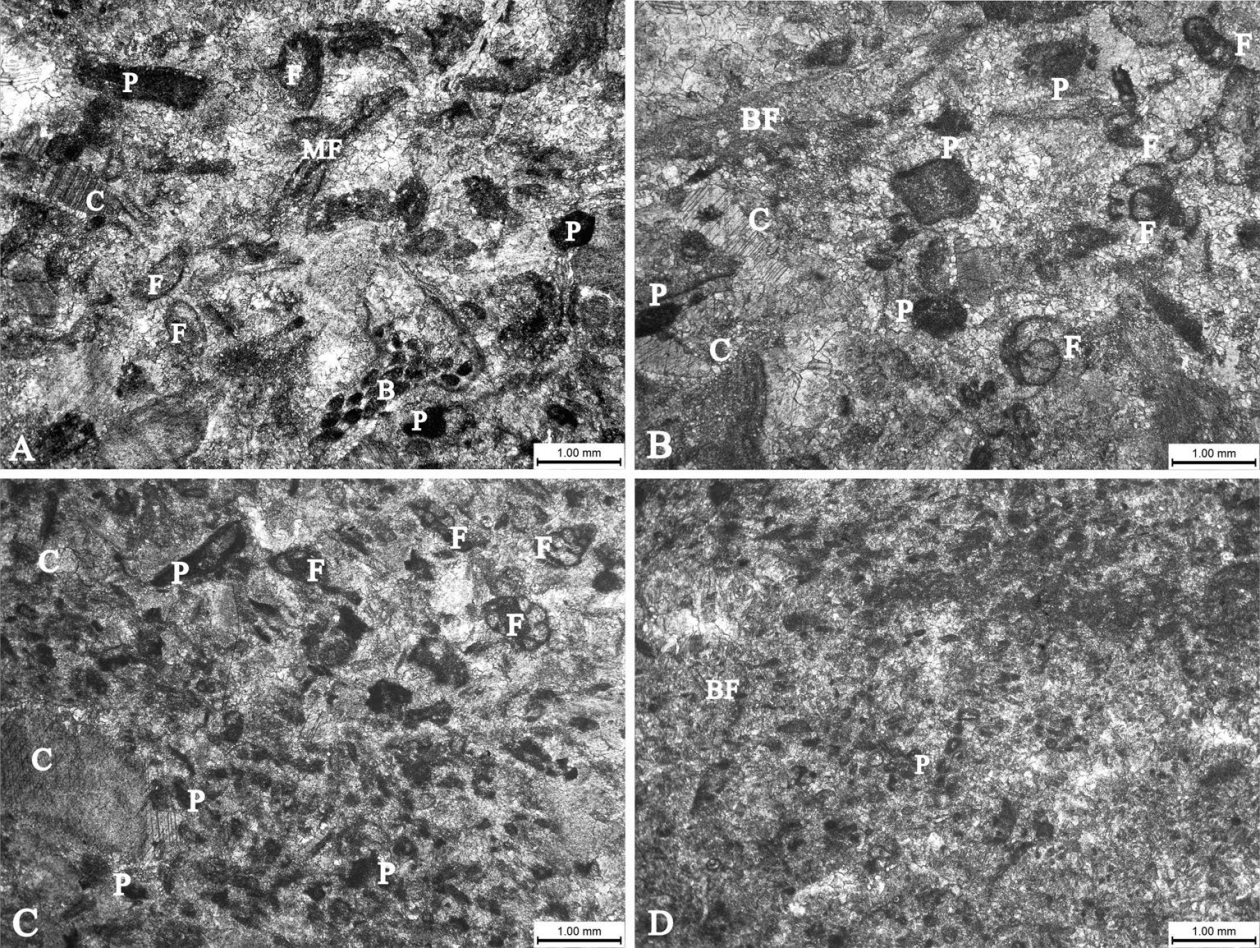
Micrographs of sediments in different positions of reef. (**A**) Calcareous bioclastic limestone, with biological particles accounting for about 70% of the debris. Abundant and diverse organisms indicate a medium-energy environment of the subtidal zone. Samples were taken from the bioclastic beach at the reef base. (**B**) Slightly larger bioclastics but lower biologic content than that in (**A**) suggest an increasing water energy. (**C**) Various bioclastic particles account for about 80% of the clastic particles contained in the calcareous bio-granular rock. The obviously small benthos indicate a low-energy environment in the subtidal zone barriered by the *Diphyphyllum* clusters. (**D**) Bioclastic grainstone is mainly composed of marl, with fine clastic particles (about 30%) and bedding. Low biomass indicates a low-energy environment of the subtidal zone. This figure is modified by the author with CorelDRAW (version 2022, and the URL link: https://www.coreldraw.com/cn/). Meaning of the letters in the figure: *C* crinoids, *BF* brachiopods, *F* foraminifera, *B* bryozoan, *P* pelletoid, *MF* mollusk shell fragment.

The Longjiangdong multi-layer reef was composed of three relatively independent, flat reef layers, suggesting three distinct periods of reef development. Diverse species were identified in the reef, with colonial coral *Diphyphyllum* contributing greatly to reef growth. *Diphyphyllum* clusters colonized in patchy form on substrates composed of bioclasts or lithic gravels (Fig. 6A). The first reef-building process was brief, ending under high-energy water conditions after a period of growing (Fig. 6B). Subsequently the hydrodynamic conditions became weaker and favorable. Then *Diphyphyllum* once again flourished. *Diphyphyllum* clumps in the unit grew closely together in strong currents, with larger and more sparse individuals than in the lower units. A relatively low energy environment was formed between the *Diphyphyllum* clusters (Fig. 6C). Subsequently, *Diphyphyllum* could only grow in a limited area of suitability due to the disturbance of high-energy water brought about by short-term sea-level rise and fall. Afterwards, the environment became more favourable and *Diphyphyllum* expanded rapidly. As a result, the upper unit of Longjiangdong coral reef was formed, in which *Diphyphyllum* individuals were slightly larger than those in the first two units. Finally, because the kinetic energy of the water continued to weaken, the plaster deposition forced the whole coral reef to stop growing (Fig. 6D).

Longjiangdong patch reef started to develop in a relatively deep water environment. *Diphyphyllum* initially colonized and expanded in favorable conditions with the increase of water energy. Then the reef-builders transitioned from a single coral species to an assemblage of *Diphyphyllum–Caninia–Lithostrotionella*. These three coral species grew independently and contribute almost equally to the structure of the reef. However, the structure and function of the coral community were not yet stable enough. It was easily influenced by the weakening hydrodynamics and the increasing sedimentation, resulting in only small patch reefs.

The Xinzhai layer reef was initialized by colonization and expansion of *Lonsdaleia* on bioclastic beach. Large coral clusters were formed in the presence of turbulent water. With the weakening of hydrodynamic conditions, an unknown branchlike organism and *Antheria* communities continued to develop separately in this area. Slender branchlike organisms expanded rapidly in these low-energy water environments until they were replaced by some individual corals as hydrodynamic energy increased. Each builder was short-lived in this layer reef, departing from the reef just at the beginning of colonization and expansion, due to rapidly changed hydrodynamic conditions.

The evolution of reef-building corals in these four reefs indicated that both the coral assemblages and coral individuals would constantly adapt to the changing hydrodynamic conditions in Langping as sea level rose and fell. Although this was a reactive adjustment of coral populations in response to long-term environmental impacts, it was clearly positive for the building and development of coral reefs.

#### Impact of disturbance on reef communities

Disturbance is a relatively discontinuous event, which is ubiquitous in nature. It may indirectly affect the composition and population structure of reef communities by changing the environmental conditions, thus affect the structure and function of reef communities, even the evolution of the reef^35^. The major disturbances evident in these Mississippian framework reefs were associated with frequent changes of water flow, and drastic changes of climate and weather. These seem to be most obvious in the Langping platform due to its small size, with more frequent environmental influence evident on the reef communities in the study area.

The most direct effect of disturbance events on coral reefs is the disruption of continuously evolving reef communities, which is common in coral reef studies. After the interruption caused by disturbances, some communities gradually recover due to the absence of continuous disturbance, or the dominant biota may be substituted by invading communities. The winner after interruption is decided by random factors to a large extent, in a ‘Competitive lottery’^36^. The conditions for the emergence of ‘Competitive lottery’ also include the need for species in a community to have similar abilities to invade discontinuities and to tolerate environmental conditions.

Certainly, low-intensity disturbance does not necessarily produce discontinuity, but medium-intensity disturbance without discontinuity could directly impact on community species diversity. According to the ‘Moderate disturbance hypothesis’, moderate disturbance is conducive to a higher level of community diversity^37^. In environmental conditions with moderate intensity of disturbance, most species will not disappear entirely. The dominant pioneer species will also be restrained by disturbance to a certain extent, so large number of species can coexist, attaining the highest diversity^35^.

**Figure 7:**
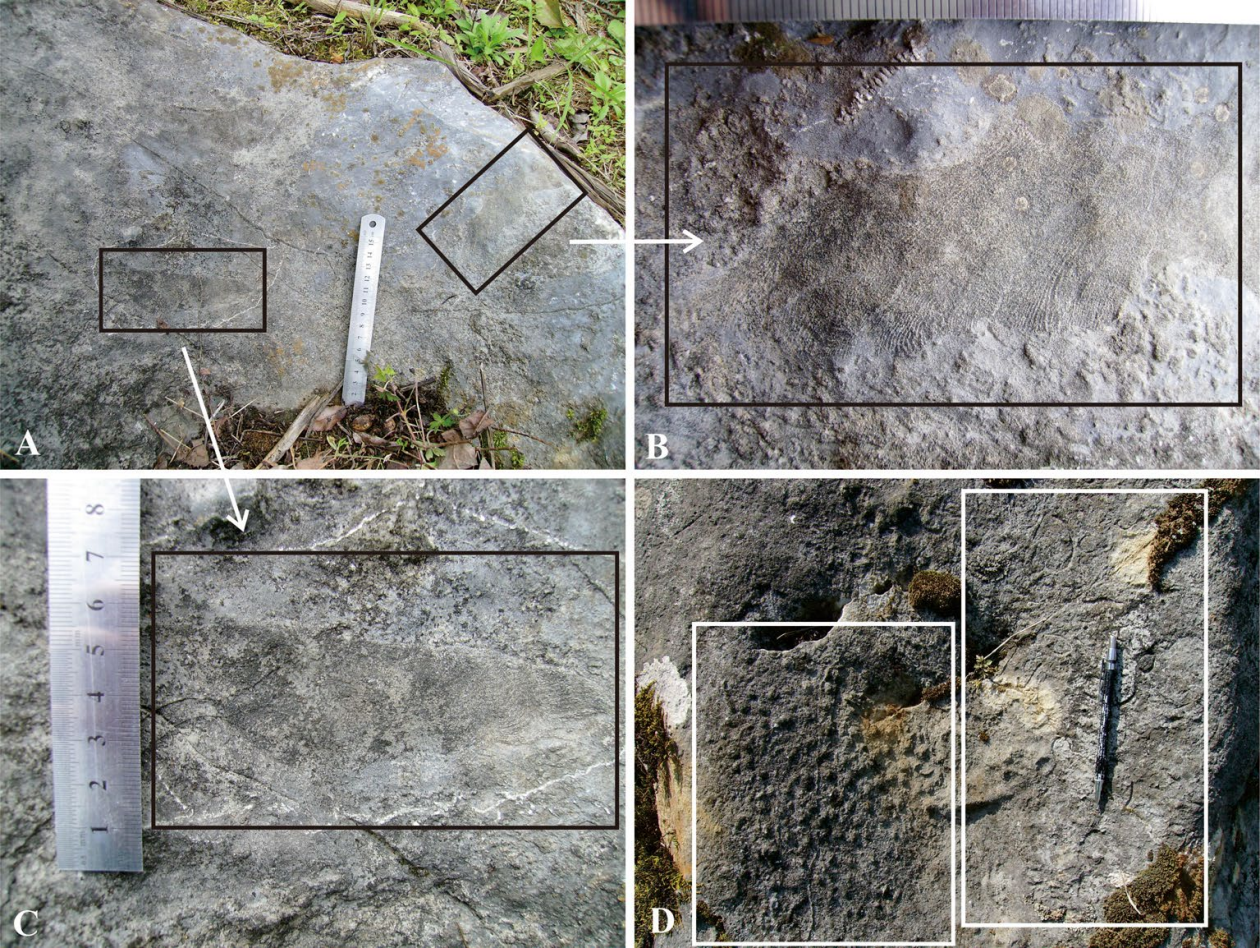
Different species occupied the discontinuity surface irregularly. (**A**) Different reef-builders colonized and grew on the same hard substrate. (**B**) and (**C**) show detailed morphology of colony corals of (**A**). (**D**) Colony corals and a large number of individual corals grew together in a limited area, indicating equal colonization on the newly formed discontinuity surface. This figure is modified by the author from field photos with CorelDRAW (version 2022, and the URL link: https://www.coreldraw.com/cn/).

The reef-builders in Langping are diverse compared with the Late Carboniferous reefs in Ziyun County^10^, which also developed in Dian-Qian-Gui Basin. More than 4 reef-building corals are identified in Xiadong reef, while 4 and 3 are in Xinzhai layer reef, Longjiangdong patch reef respectively. These reef-building corals, mostly *Diphyphyllum*, *Lithostrotion*, *Siphonodendron* and *Lonsdaleia*, were distributed irregularly in the reefs. Their ecological niche and function were likely similar and none of them was obviously dominant in the community (Fig. 7). This is in line with ‘Competitive lottery’ theory and the ‘Moderate disturbance hypothesis’.

The stability of a classical reef ecosystem includes the ability to withstand external disturbances and the ability to return to its original state once the disturbance is removed^37,38^. It is generally accepted that communities with high diversity are always more stable although ecosystem stability is not absolutely correlated to biodiversity^35^.

There have been no reef-building corals with strong resistance and rapid recovery ability in the communities in Langping. None of these corals succeeded in developing into dominant species that can build reef shelves, which made the reefs in Langping mostly appear in the form of small patch reefs or reef layers. However, formation of the large reef in Xiadong Village, patch reef in Longjiangdong Village, and layer reef in Xinzhai Village were all related to their relatively high diversity of reef-building corals. Compared with the situation where only one reef-building organism dominated the Bianping large coral reef, Wengdao large phylloid algal reef and *Ivanovia* cf. *manchurica* patch reefs in Ziyun County^10^, Guizhou province, the different coral assemblages in Langping area could effectively adapt to changing hydrodynamic conditions and maintain reef growth.

Species diversity increased by disturbance stabilized the ecosystemas shown during the construction of coral reefs in Langping.

#### Effects of non-reef-builders on reef-building corals

Besides reef-building corals, there were a large number of reef-dwellers and off-reef organisms in the study area. Reef-dwellers referred to the species that didn’t directly contribute to reef growth in the community, mainly including various benthos and algaes^39^. Off-reef organisms are not part of the reef-building community, but also play an important role in participating in energy flow and providing organic matter to the reef ecosystem^40^.

Common reef-dwelling organisms include crinoids, brachiopods, gastropods, various algae, foraminifera, bryozoans and individual corals. Crinoids were overwhelmingly dominant in numbers in the reef samples studied here.

Carboniferous echinodermata in Guangxi Province reached its peak in Middle-to-Late Mississippian. In terms of amount and distribution, thick limestone with echinodermata debris in the carbonate platform were often dominated by crinoids^41–46^. The large number of crinoids in Langping excluded other metazoans and restricted the development of benthic reef-builders in Late Viséan-Serpukhovian in Langping, leading to poorly developed reef-building communities.

**Figure 8:**
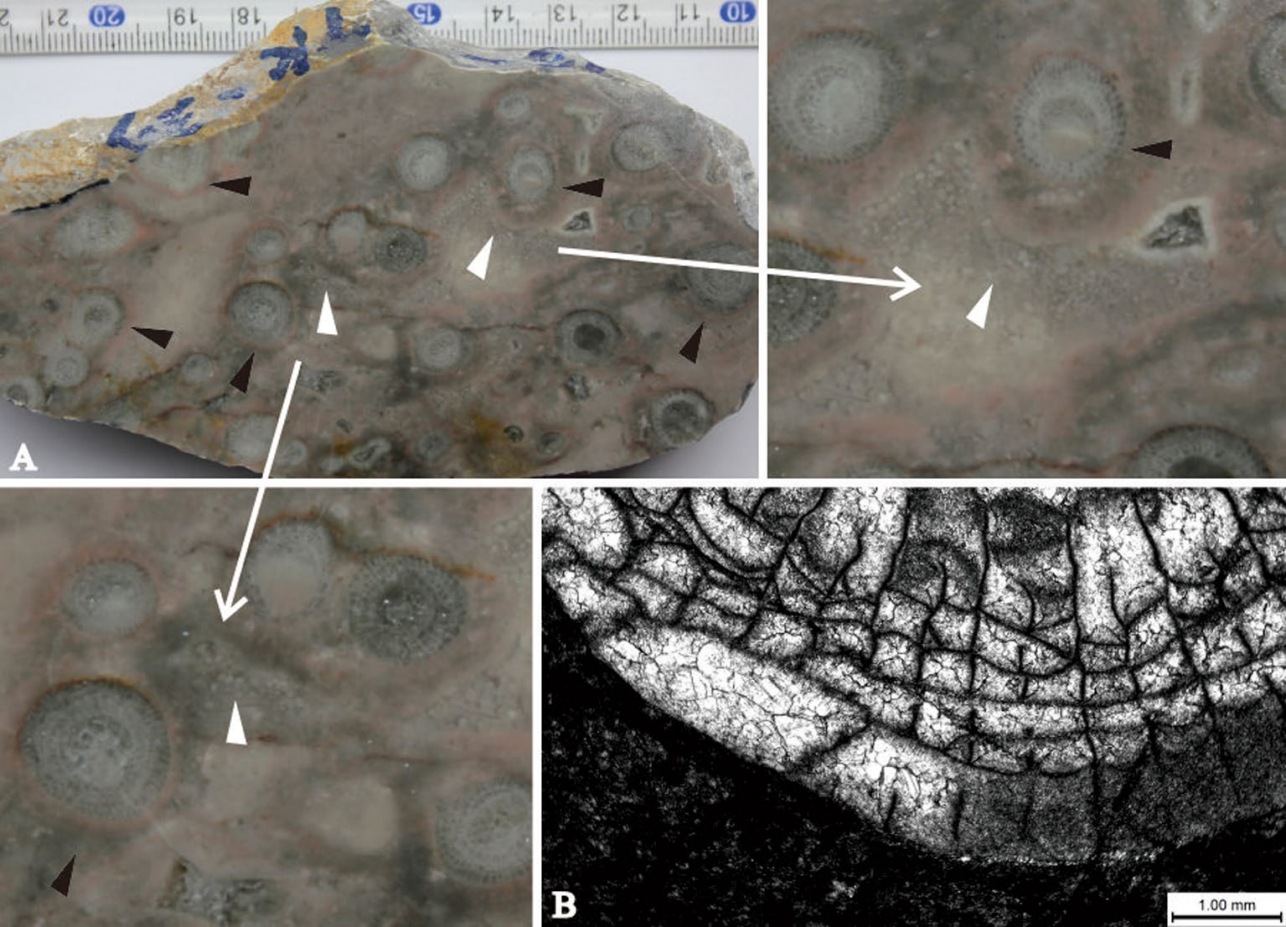
Micrographs of microbes and algaes. (**A**) Encrustations (indicated by black arrows) with distinct thickness around coral clusters formed by microbe and algal mats through bonding mud. The encrustations were formed before the clastic deposition (indicated by white arrows), showing the corals were living then. Microbes and algaes inside of the dense coral clusters had little impact on corals. (**B**) Single polarized micrograph showed clear and smooth boundaries of coral individuals without encrustation or drilling hole made by microbes or algaes. Few corals surrounded by bonding algaes could be observed in Langping, indicating that algaes were poorly developed between coral clusters. This figure is modified by the author from field photos with CorelDRAW (version 2022, and the URL link: https://www.coreldraw.com/cn/).

Microorganisms and algaes had limited success in stablishing on the moving clastic beach in frequently disturbed water. There has not been obvious evidence of extensive “algal turf” in the coastal area of Langping platform. Only a few corals bonded by algal mats were observed^47^ (Fig. 8). In addition to their significant contribution to primary productivity, macroalgae were considered to play an important role in two aspects of coral reef ecosystems. One was to promote reef construction by its own binding and consolidation^48,49^. The other was to create a good condition for zoobenthos larvae to dwell and develop, thereby improving species diversity^50^. The limited productivity of algae in Langping constrained coral reef trophic inputs, which may then have limited populations of dependent metazoans. As a result, algaes and other metazoans were unable to achieve a variety of reef-building patterns, such as bonding, bounding, entanglement^51,52^. The reef framework in the study area was not stable in the presence of strong water flow, and the biological communities could not deal with frequent environmental changes, which were directly related to poor development of calcareous algae.

#### Influence of coral morphology on reef development

The accumulation of reef structure had obvious impact on communities. Large reef structures could support abundance and diverse biota by modifying local environments and creating diverse conditions. Consequently the reef-building communities thrived between disturbances, stabilizing reef construction. In terms of large reef, the framework-building corals would play a key role in reef construction regardless of which kind of patterns was adopted. Therefore, reef-building corals with large size, rapid growth vertically, and strong resistance would become the biggest contributors to reef frame construction.

**Figure 9:**
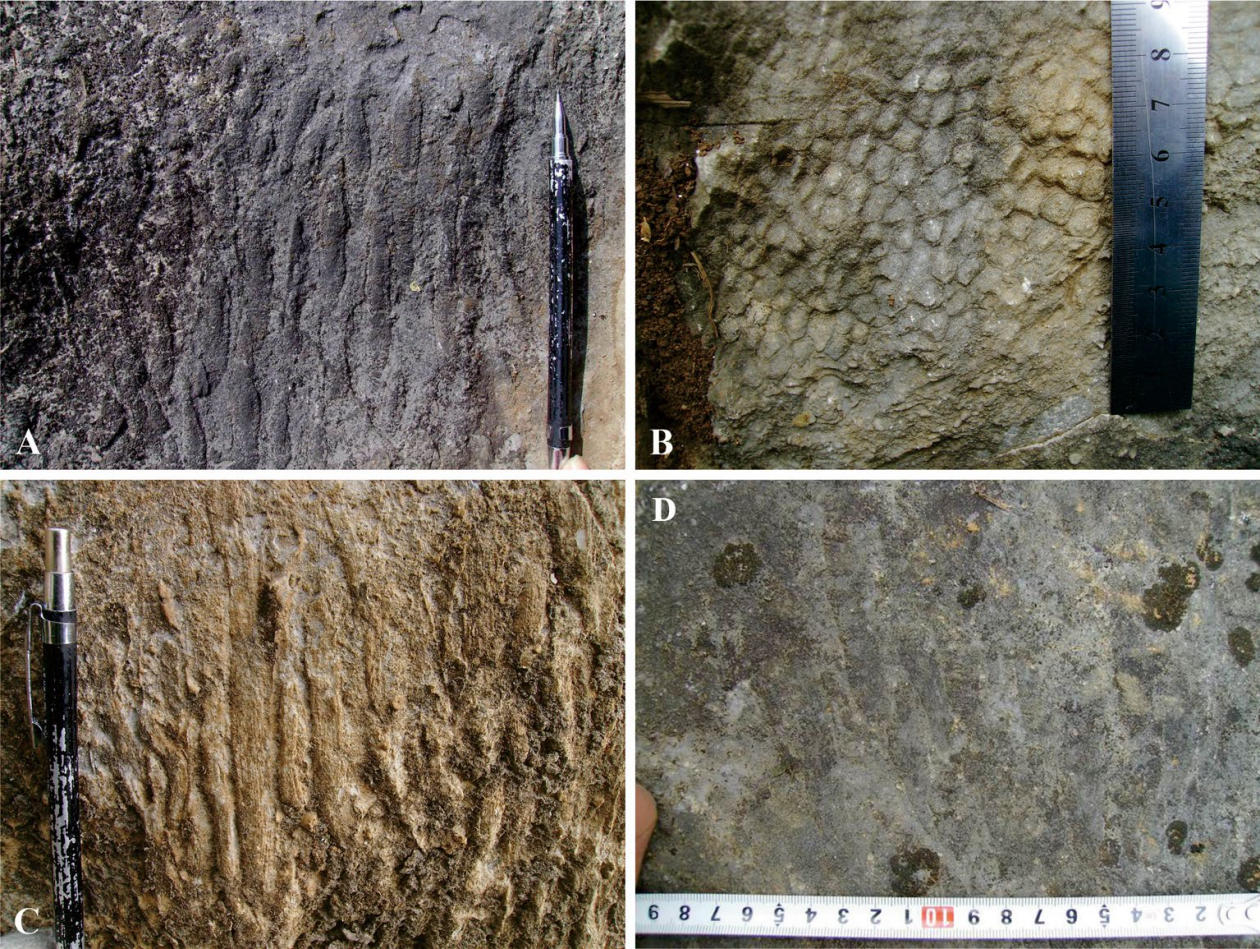
Main reef-building corals in the study area. (**A**) *Diphyphyllum,* (**B**) *Lithostrotion,* (**C**) *Siphonodendron,* (**D**) *Lonsdaleia*. (**A**) Rapidly grew clusters of main reef-building corals. The strong individuals are packed tightly when growing to support each other. This figure is modified by the author from field photos with CorelDRAW (version 2022, and the URL link: https://www.coreldraw.com/cn/).

The main reef-building corals in Langping were composed of *Diphyphyllum*, *Lithostrotion*, *Siphonodendron,* and *Lonsdaleia*, etc., being the dominant builders. These corals were similar in morphology such as cluster colony, thick and strong skeleton, and densely packed individuals (Fig. 9), which enabled them to resist water flow. At the same time, the upright colonies were adaptable to relatively calm water, being insensitive to mud deposition. The ecological characteristics of the Langping corals matched the gently sloping environment, the deep water environment and the rapidly changing energy of the currents. These cluster corals were able to colonize hard substrates and expand rapidly, thus altering the surrounding environment. The visible carbonate uplifts were formed with a large amount of benthos grouped into reef-building communities. These distinct uplifts constructed by coral clusters in different water conditions are composed of coral reefs of different sizes and appearance in the study area.

The complex and diverse local environments formed by large coral reefs can significantly increase benthic populations and improve reef species diversity. As a result, the nutrient flow in the community becomes complicated, and nutrients could be recycled effectively by reducing loss caused by water flow. Therefore, the overall productivity of large coral reef communities was always high. Complex trophic structure satisfied most of the benthos in the community with sufficient nutrients and inorganic salts.

The morphology of reef-building corals in Langping enabled them to become predominant species in various water environments, which promoted the continued domed growth of coral reefs and facilitates the development of reef-building communities that form a variety of reefs. It suggests that the morphology of reef-building corals was a key prerequisite for reef development.

In conclusion, coral reef communities are always constrained and influenced by environmental conditions. However, the ecology of the inhabitants is also an important factor in the formation of coral reefs.

## Conclusions

Based on the environment and ecology analysis of the four Late-Mississippian reefs in Langping, new understanding of the influencing factors and evolutionary mechanism of reef-building process are summarized as follows.

1. The climate, sea-level conditions in Late Viséan-Serpukhovian and the tropical ocean current flowing through the Langping area ensured that the reef-building corals could continue to survive and build reefs under unfavorable geological background.
2. The gentle slope of Langping platform provided the necessary conditions for the benthic communities. And water environmental factors determined the general appearance of reefs in the study area.
3. The adaptability of the morphological characteristics of reef-building corals to the variable hydrodynamic conditions is of positive significance to the continuous construction of coral reefs.
4. The construction process of reefs has confirmed that disturbance can increase the species diversity of reef communities and enhance the stability of ecosystems, and other biological factors also have a certain impact on the development of reefs.

## Data Availability

These materials were stored in the Department of Geology, Northeastern University, Shenyang, China. The datasets used during the current study are available from the corresponding authors on reasonable request.
